# COVID-19 Pandemic Causing Depression in Different Sociodemographic Groups in Saudi Arabia

**DOI:** 10.3390/ijerph18136955

**Published:** 2021-06-29

**Authors:** Hana Sonbol, Hadil M. Alahdal, Rasis A. Alanazi, Khawla Alsamhary, Fuad Ameen

**Affiliations:** 1 Biology Department, College of Science, Princess Nourah Bint Abdulrahman University, Riyadh 11671, Saudi Arabia; HSSonbol@pnu.edu.sa (H.S.); hmalahdal@pnu.edu.sa (H.M.A.); 2 Psychology Department, College of Education, Princess Nourah Bint Abdulrahman University, Riyadh 11671, Saudi Arabia; RAAlanazi@pnu.edu.sa; 3 Department of Biology, College of Science and Humanities in Al-Kharj, Prince Sattam Bin Abdulaziz University, Al-kharj 11942, Saudi Arabia; k.alsamhary@psau.edu.sa; 4 Department of Botany & Microbiology, College of Science, King Saud University, Riyadh 11451, Saudi Arabia

**Keywords:** COVID-19, depression, pandemic, precaution measures, mental health, Saudi Arabia

## Abstract

COVID-19 disease was announced as a global pandemic in March 2020 by the World health organization (WHO). Saudi Arabia was among the first countries to enforce restriction measures such as closing schools, remote working, and a travel ban. We aim to evaluate the impact of the COVID-19 pandemic on people’s depression in Saudi Arabia. A cross-sectional online survey of 1109 participants was conducted during the curfew between 18th of May and 11th of June 2020. An online questionnaire included questions about the commitment to follow the precautionary measures, knowledge on COVID-19, and depression. Depression was assessed with the Impact of Event Scale-Revised method. Females, unmarried individuals, elderly persons, parents of young children, unemployed, and small families were more likely to be depressed. Education level did not explain the differences in depression. However, the more knowledge the participants had about COVID-19 the better they followed the restrictions. A regression analysis revealed that the commitment of a person to follow the restrictions increased his/her depression symptoms. Attention should be paid to different groups of people in future psychiatric planning.

## 1. Introduction

The coronavirus disease (COVID-19) first emerged in December 2019 in China and caused a global health pandemic [1,2]. The total number of COVID-19-infected people has been accelerating, and the death count is exceeding previous Middle East respiratory syndrome coronavirus (SARS-CoV) epidemics [3]. In Saudi Arabia, the first coronavirus infection was reported in March 2020 [4]. More than 470,000 people have been infected in Saudi Arabia as of June 2021.

To prevent the transmission of COVID-19 infection, significant intervention such as physical distancing and the use of face masks is widely recommended [5,6,7]. Saudi Arabia was one of the first countries that imposed strict measures including the limiting of outdoor activities, closing schools, minimizing social contacts, and banning mosque prayers [8]. The entire country was quarantined, and curfew was legislated in big cities.

The restrictions may have caused serious impacts on the mental health of the public. The sudden change in people’s routine can predispose one to depression. COVID-19 as a new emerging virus with unique features and high infectious rates predispose people to high levels of stress. COVID-19 news in all media, the numerous hypotheses of its mode of transition and consequences, and the fear of getting the infection personally for family members can all be predisposing factors for depression. A recent study in Saudi Arabia indicated that about one-third of individuals studied had moderate to severe depression during the COVID-19 pandemic [9]. Younger people, people spending too much time thinking about the outbreak, and healthcare workers were at high risk of mental illness in China [10]. In Saudi Arabia, it is not known how and to what extent the epidemic is affecting different sociodemographic groups of people. Such studies are crucial to help determine general mental health status and anticipate possible mental disorders.

Our aim was to assess the level of depression during the COVID-19 pandemic in different sociodemographic groups and how following the precautionary measures affected depression symptoms. We measured the depression burden in adults living in Saudi Arabia during the period of curfew using a questionnaire and examined the relationship between participants’ depression level and protecting factors such as commitment to follow the precautionary measures, education level, and family circumstances. Preparedness to face the virus-related mental health outcomes will help to treat the issue at an early stage.

## 2. Research Methodology

### 2.1. Study Design

A snowball sampling recruitment method was used to recruit adults living in Saudi Arabia (18–55 years old) between 18th of May and 11th of June 2020. The participants were recruited through WhatsApp chains starting from the researchers who asked their contacts first. Participants (*n* = 1109) completed an online survey through Google Forms in the Arabic language. It took about five minutes to complete, and communication between the researchers and the participants was possible. The participants had the freedom to stop whenever they wanted. Expedited ethics approval was obtained from the Institutional Review Board of the Princess Nourah bint Abdulrahman University (PNU) (20-0215). All respondents provided informed consent. 

The public’s psychological response and awareness about precautionary measures during the pandemic of COVID-19 was assessed using a cross-sectional survey design. Correct answers were given 3 points, while not knowing the answer received 2 and wrong answers received 0. The questionnaire was adopted from studies where it was pre-tested and validated [10,11,12]. The questionnaire about depression was from the Center for Epidemiologic Studies Depression Scale (CES-D) excluding three items [13]. The CES-D score ranged between 0–51 points, and higher scores indicated more severe depressive symptomatology [13]. 

All methods followed the guidelines of the National Committee of Bioethics (NCBE), Saudi Arabia. The questionnaire consisted of questions covering the following areas: (1) Sociodemographic information, (2) commitment to follow precautionary measures, and (3) feelings of depression. 

The maximum score for the commitment to follow precautionary measures was 28 and for depression was 51, with higher scores indicating more severe depressive symptoms. The reliability and validity of the scale was assessed using a pilot test with 94 participants. Scale reliability was tested using a Cronbach’s Alpha (α) and Spearman–Brown coefficient (0.90). The depression scale demonstrated acceptable internal consistency (α = 0.89). The assessment was based on previous studies [14] where α > 0.70 is acceptable in social science research.

### 2.2. Statistical Analysis

One-way ANOVA followed by the least significant difference test (LSD) were used to analyze the differences in the variables of precautionary measures and depression between the sociodemographic groups. The Pearson correlation was calculated, and a regression analysis was carried out between the variables of precautionary measures and depression. *p* < 0.05 was considered to be significant. SPSS Statistic 21.0 ^®^ (IBM SPSS Statistics, Armonk, NY, USA) was used.

## 3. Results

### 3.1. Sociodemographic Characteristics

A total of 1109 participants completed the survey. The majority of respondents were female (74%, *n* = 824) and married (72%, *n* = 793). About half were under 45 years old, where 26% (*n* = 286) were 26–35 years old and 24% (*n* = 261) were 36–45 years old. Participants were mostly well educated, 65% (*n* = 719) had a bachelor’s degree, and 21% (*n* = 233) had a higher degree. About half (47%, *n* = 516) were employed either in the private or government sectors or were entrepreneurs, while about one-third (32%, *n* = 351) were unemployed. Most (91%, *n* = 1013) belonged to families with between three and six members and about two-thirds had children (69%, *n* = 769). Most of the participants were from the Western Region (58%, *n* = 642) and the Central Region (35%, *n* = 391). The participants were more educated than the average population in Saudi Arabia where 23% of the population has a bachelor’s degree or were at a respective level [15]. Old people were less represented than in Saudi Arabia where the demographic profile is as follows: 0–14 years: 24.8%, 15–24 years: 15.4%, 25–54 years: 50.2%, 55–64 years: 5.9% and 65 years and over: 3.6% [16].

### 3.2. Sociodemographic Groups and Precautionary Measures

Most of the participants always covered their mouth when coughing and sneezing (83%) and washed their hands with soap and water (77%) (Table 1). About half always avoided the sharing of utensils, washed their hands immediately after coughing or sneezing, and wore masks. About one-third (31%) of the participants felt that the COVID-19 pandemic has caused too much unnecessary panic while 77% avoided leaving their homes.

The maximum score for precautionary measures observed was 27 and the minimum was 6. A significant difference (*t*-test, *p* < 0.05) in the scores of males (19.84, SD = 3.91) and females (21.17, SD = 3.82) was found (Table 2). Marital status had no effect, as no significant difference between married and unmarried participants was found. The extent of knowledge of Covid-19 presented an effect, where participants with much knowledge had a significantly higher score (21.08, SD = 3.75) than those with little knowledge (19.40, SD = 4.27). Higher education increased the commitment to precautionary measures. Undergraduate students (20.78, SD = 3.85) and post-graduates (21.36, SD = 3.42) were significantly (ANOVA, LSD, *p* < 0.05) more committed to the precautionary measures than the participants with lower education (high school participants, score 20.25, SD = 4.55). The participants who had felt concerned about the disease for three hours or more (22.18, SD = 3.60) were more likely to respond to precautionary measures than the less concerned ones (less than one hour, score 20.27, SD = 3.8) (Table 2).

### 3.3. Sociodemographic Groups and Depression

About half of the participants were relatively relaxed, where 52.57% had not lost their appetite, 56% felt depressed less than one day a week, 52.57% felt hopeful about the future, 68.53% did not feel that their life was a failure, 59.15% thought that people are unfriendly less than once a day, and 62.58% cried less than once a day (Table 3). About half of the participants (51.94%) felt sad, although they received support from their family and friends.

A significant difference was observed in the CES-D scores between married and unmarried participants, with those unmarried having higher scores (17.26, SD = 9.78) than those married (14.56, SD = 10.11) (Table 4). Females (16.25, SD = 10.23) had significantly greater scores than males (12.69, SD = 9.19). The CES-D score was not affected by the extent of knowledge of Covid-19. Participants who were concerned about the COVID-19 epidemic, spending three hours or more following the news, had higher scores than the less concerned ones. People under 55 years had higher scores (14.8–17.2) than those older than 55 years (12.21, SD = 9.97). Education had no significant effect. The number of children had a significant effect, where persons with children older than 16 years (13.45 SD = 10.06) had lower scores than people with younger children (15.84 SD = 10.23) or without children (16.34, SD = 9.72) (LSD test). Families with less than two members were significantly more depressed (17.35, SD = 10.39) than families with more than six members (14.55, SD = 9.67). Unemployed participants were significantly more depressed (16.18, SD = 10.25) than retirees (M = 12.83, SD = 9.90). The time spent engaging with COVID-19 news was reflected in the level of depression, where participants who spent more than 3 h daily had more depression (19.59, SD = 11.02) than the ones who spent one hour (13.89, SD = 9.64) or less than three hours (15.90, SD = 9.63).

### 3.4. The Relation between the Precautionary Measures and Depression

CES-D score correlated most strongly with anxiety (*r* = 0.44) and the next strongly with wearing a mask (*r* = 0.12) (Table 5). The total scores of precautionary measures taken by the participants positively correlated with CES-D score (*r* = 0.22).

A significant regression equation was found (F (1, 1107) = 59.37, *p* < 0.01) between total precautionary score and CES-D score with anR^2^ of0.051 (Table 6). CES-D score is equal to 2.61 × total precautionary score + 0.61 indicating that the participants’ CES-D score increased 0.61 for each increase in precautionary measures. 

## 4. Discussion

The level of following good disease preventing practices was moderate to high in our study. This result was expected, as public awareness has been improved in Saudi Arabia especially with the MOH adequately updated information presented on all media channels.

Only about half of the respondents washed their hands with water and soap and covered their mouth when coughing or sneezing. About one-quarter of the participants in our study did not always, or even most of the time, avoid sharing utensils during meals. The lack of precautionary practice during meals is probably accelerating the transmission. This has been observed previously, as many disease cases originate from sharing meals [17]. Moreover, asymptomatic individuals cover their mouths when coughing and sneezing more often than symptomatic individuals [18]. The situation during the COVID-19 pandemic may worsen because of the social nature of family-oriented Saudi people with many gatherings and family activities. Thus, maintaining precautionary measures is essential. Moreover, recommendations and updates from local authorities and WHO increases people’s awareness and helps people to follow precautionary measures [19,20,21].

In this study, married and elderly people as well as members of large families obtained lower CES-D scores measuring depression. These factors were thus protective against depression. Females appeared to be more depressed than males. This was not surprising since, according to the WHO, women are susceptible to common mental disorders such as depression and anxiety [22]. The age of children was found to be correlated with depression symptoms; the younger the children were, the more likely their parents had depression. This has been reported previously; parents of young children declare more depressive symptoms than parents of adult children [23]. In our study, unmarried individuals were more depressed than married couples. Regarding age, we found that less depression was associated with older people; participants above 55 years were less depressed. The two latter observations contradict previous studies that mostly show more depression in married and older individuals. Marriage was attributed to the great number of responsibilities [24,25]. Older people, in turn, are thought to have greater risk for depression because of their social disconnection and isolation feelings [26,27]. Our different findings might be explained by the family-oriented nature in Saudi Arabia. The conventional norms of the Saudi society also protect elderly people who mostly live with their children and not by themselves, and seldom in care houses. Additionally, bigger families protected against depression, as family members may provide support to each other.

It seems that the commitment to follow precautionary measures increased depression symptoms because a positive, although weak, correlation between the scores for CES-D and total precautionary measures was observed. The maximum score for depression in the depression scale CES-D is 51, and the cutoff score of 16 indicates a risk for clinical depression [28]. The regression analysis of our data indicated that the participants’ CES-D score was increased by 0.61 units with each increase in the precautionary measures. Thus, it seems that the risk for depression is relatively high in small families, for females, unmarried, unemployed, individuals younger than 35 years old, or with no children. All these groups obtained a score higher than the cutoff of 16 in our study. This interpretation must be done with caution because the explanatory power of the regression analysis was relatively low, and the precautionary measures explained only 5% of the variation in CES-D score.

Behind the commitment to follow the precautionary measures was good knowledge about COVID-19, time spent on COVID-19 information, and high education, which were positively correlated with the commitment to follow precautionary measures. A recent study revealed that individuals with higher education had higher awareness about the precautionary measures of SARS virus [29]. Moreover, females appeared to follow precautionary practices better than males, which has been observed in some previous studies [29,30,31].

Lockdown and depression seem to be strongly linked. Despite that the curfew due to COVID-19 pandemic is different, it involves locking people in their houses and restricting their movements. Several studies have associated individual’s lockdown with depression [32,33]. Studies have shown that limited outdoor activities can result in depression [34,35]. Outdoor activities are linked with physical exercise, which is known to improve mental health [36,37,38]. One important factor is light, as it provides signals to the brain to maintain circadian rhythm, which is involved in the sleep/wake cycle and is linked to the secretion of several mood and happiness hormones such as melatonin, cortisol, and serotonin [39,40,41]. Additionally, light exposure is linked to the maintenance of vitamin D levels, as lower levels of vitamin D are associated with depression [42,43].

The limitations of the research design have an effect on the reliability of the results. First, the survey lacks pre-COVID and post-COVID results about depression, and therefore the results must be interpreted with caution. However, several other recent articles report the increased depression symptoms and a number of psychological disorders during COVID-19 pandemic [44,45,46,47]. Moreover, the relation of the commitment of the individual to follow precautionary measures and depression symptoms can be assessed as reliable in our study. Second, the sociodemographic profile of the participants did not follow the actual profile of Saudi Arabia. Women, educated, and young people were overrepresented due to the recruiting process. However, our result that women felt more depression symptoms than men has been observed elsewhere, as reviewed [48]. One more limitation is that our cross-sectional design does not allow us to make any causal inferences. A web-based survey and snowball sampling recruitment method also create possibilities for selection bias. We were also not able to assess individuals’ psychological condition before the pandemic. However, we suggest that the results show the potential of increased clinical depression cases caused by the pandemic.

## 5. Conclusions

We found a positive relationship between precautionary measures and depression. We also found that vulnerable individuals including elderlies and guardians with bigger families had relatively low depression levels in Saudi Arabia, which may be a benefit of a family-oriented lifestyle. It is recommended for governments and health authorities to not only provide masks, soaps, and disinfectants, but also support for mental health. Online awareness programs initiated by the government, media channels, and universities are important to increase people’s knowledge about the situation and guide them through the pandemic. Free consultation services and a trauma focused-cognitive behavior therapy can be launched online for people in need. Furthermore, follow-up procedures should be taken to ensure the well-being of people. Our findings can be used to formulate psychological interventions to improve mental health and psychological resilience during the COVID-19 epidemic and to improve the precautionary measures practice.

## Figures and Tables

**Table 1:** Percentage of participants (*n* = 1109) following different precautionary measures. Response alternatives: 1 = never; 2 = occasionally; 3 = sometimes; 4 = most of the time; 5 = always.

Precautionary Measures	Response %				
	1	2	3	4	5
Q1: Covering mouth when coughing and sneezing	0.2	1.6	10.8	4.0	83.4
Q2: Avoiding sharing of utensils	7.6	6.1	12.0	24.0	50.3
Q3: Washing hands with soap and water	0.0	1.7	3.1	17.9	77.3
Q4: Washing hands immediately after coughing, rubbing nose, or sneezing	5.5	35.3	3.3	6.8	49.1
Q5: Wearing mask regardless of the presence or absence of symptoms	11.3	11.6	18.6	3.3	55.2
Q6: Feeling that other people are too anxious about COVID	9.0	16.4	31.0	25.2	18.4
Q7: Hours stayed at home	<9 h	10–19 h	>19 h		
	4.0	18.4	77.6		

**Table 2:** Participants (*n* = 1109) committed (mean, SD) to the precautionary measures in different sociodemographic groups and the *p* value of ANOVA indicating the significant effect of the group.

Variable	Group	*N*	Mean	SD	*p* Value
Gender	Male	285	19.84	3.91	0.010
	Female	824	21.17	3.82	
Marital Status	Married	793	20.75	3.95	0.32
	Unmarried	316	21.01	3.7	
Knowledge of COVID-19	Much	939	21.08	3.75	0.01
	Little	170	19.4	4.27	
Age	18–25	165	20.5	3.9	0.1
	26–35	261	20.93	3.57	
	36–45	286	20.9	3.82	
	46–55	220	21.27	4.01	
	>55	177	20.29	4.2	
Condition as dependent	No kids	340	20.63	3.9	0.4
	<16 years	461	21	3.84	
	>16 years	308	20.78	3.92	
Education	High school	157	20.25	4.55	0.05
	Undergraduate	719	20.78	3.85	
	Post-graduate	233	21.36	3.42	
Family Size	≤2	96	20.79	4.6	0.78
	3–5	528	20.91	3.73	
	≥6	485	20.74	3.89	
Employment Status	Student	92	20.54	4.28	0.38
	Non-employed	351	21.11	3.79	
	Retired	150	20.63	4.47	
	Employed	516	20.73	3.68	
Hours spent on COVID-19 news daily	≤1	655	20.27	3.81	0.01
	2–3	269	21.23	3.97	
	≥3	185	22.18	3.6	

**Table 3:** Percentage of participants feeling different depression symptoms. Response alternatives: 1 = not at all (less than in one day a week); 2 = sometimes (in one or two days); 3 = every now and then (3–4 days), 4 = all the time (5–7 days).

Questions	Not at All (Less Than One Day)	Sometimes (a Day or Two)	Every Now and Then (3–4 Days)	All the Times (5–7) Days
	%	%	%	%
Q1. I get upset from things that normally do not upset me	37.06	7.03	44.72	11.18
Q2. I feel annoyed with things that do not annoy me usually	41.03	8.03	42.20	8.75
Q3. I lost my appetite	52.57	29.13	14.97	3.34
Q4. I feel sad despite of my family and friends support	51.94	5.14	34.45	8.48
Q5. I feel good	48.96	27.77	16.59	6.67
Q6. I feel depressed	56.00	5.41	32.46	6.13
Q7. I feel that every task I am doing is an effort	32.01	36.97	19.84	11.18
Q8. I feel hopeful about the future	44.27	31.74	17.22	6.76
Q9. I feel that my life is a failure	68.53	18.67	9.65	3.16
Q10. I feel scared	42.29	33.00	17.31	7.39
Q11. I cannot sleep at night	45.09	27.59	16.77	10.55
Q12. I feel happy	30.66	39.13	22.63	7.57
Q13. I talk less than usual	34.08	32.64	25.61	7.66
Q14. I feel lonely	48.96	24.35	17.13	9.56
Q15. People are unfriendly	59.15	23.44	12.53	4.87
Q16. I enjoy my life	39.68	29.58	22.90	7.84
Q17. I had crying spells	62.58	22.54	11.00	3.88

**Table 4:** The CES-D score (mean, SD) for depression in different sociodemographic groups and the *p* value of ANOVA indicating the significant effect of the group.

Variable	Group	No. Participant	Mean	Std. Deviation	T-Value	Significant
Gender	Male	285	12.69	9.19	5.47	0.01
	Female	824	16.25	10.23		
Marital Status	Married	793	14.56	10.11	4.05	0.01
	Unmarried	316	17.26	9.78		
Knowledge of COVID-19	I know a lot	939	15.27	10.24	0.51	0.61
	I have a general knowledge	170	15.69	9.25		
Age	18–25	165	17.21	9.46	6.61	0.01
	26–35	261	16.27	9.73		
	36–45	286	15.72	10.11		
	46–55	220	14.82	10.52		
	>55	177	12.21	9.97		
Conduction as dependent	No kids	340	16.34	9.72	7.76	0.01
	16 years old child or younger	461	15.84	10.23		
	16 years child or older	308	13.45	10.06		
Education Level	High school or less	157	14.92	10.21	1.2	0.3
	Undergraduate	719	15.67	10.14		
	Graduates	233	14.57	9.85		
Family Size	≤2	96	17.35	10.39	3.7	0.05
	3–5	528	15.68	10.36		
	≥6	485	14.55	9.67		
Work Status	Student	92	15.58	9.08	3.98	0.01
	Non-employed	351	16.18	10.52		
	Retired	150	12.83	9.9		
	Employed	516	15.44	9.93		
Time spent on COVID-19 news daily	≤1	655	13.89	9.64	24.54	0.01
	2–3	269	15.9	9.63		
	≥3	185	19.59	11.02		

**Table 5:** Pearson correlation coefficient (*r*) between the precautionary measures and the CES-D score describing depression (*n* = 1109, *p* < 0.05 when *r* > 0.06).

Precautionary Measures	Pearson Correlation Coefficient (*r*)
I cover the mouth when coughing and sneezing	0.003
I avoid sharing utensils	0.061
I was my hands with soap and water	0.043
I wash my hands immediately after I sneeze or after I touch my nose	0.038
I wear a mask regardless of the symptoms	0.115
I feel anxious about the spread of COVID-19	0.447
The average hours spent at home	0.015
Total scores for precautionary measures	0.226

**Table 6:** Regression analysis between the total scores of precautionary measures and CES-D score.

	Sum of Squares	DF	Mean Square	F	*p*	R^2^
Regression	6235.69	1	6235.69	59.37	0.01	0.051
Residual	116,275.86	1107	105.04			
	**Unstandardized Coefficients**	**Standardized Coefficients**		**T**		***p***
	**B**	**SE**	**Beta**			
Constant	2.61	0.68		3.84		0.01
Precautionary measures	0.61	0.08	0.23	7.70		0.01

## Data Availability

All data related to this manuscript is incorporated in the manuscript only.
